# Multiferroics: a beautiful but challenging multi-polar world

**DOI:** 10.1093/nsr/nwz093

**Published:** 2019-07-31

**Authors:** Ce-Wen Nan, Jun-Ming Liu

**Affiliations:** 1 School of Materials Science and Engineering, Tsinghua University, China; 2 Laboratory of Solid State Microstructures and Department of Physics, Nanjing University, China

Multiferroics simultaneously possess two or more primary ferroic orders, such as magnetic, ferroelectric, ferroelastic and ferrotoroidic orders. Cross-coupling among these multi-ferroic orders would result in new properties, such as a magnetoelectric effect that enables controlling magnetism using an electric field or controlling polarization remotely using a magnetic field. Since their discovery in the 1960s, multiferroic materials have constantly attracted attention and, especially since the 2000s, research on multiferroics has grown apace. The observation of room-temperature multiferroicity in BiFeO_3_ thin films has driven significant research activity in single-phase multiferroics. The observation of the giant magnetoelectric effect (i.e. magnetic-field-induced large polarization change) in multiferroic composite materials has also aroused considerable interest. With advances in film-deposition techniques, multiferroic heterostructures have become a topical subject and there have been remarkable developments in the room-temperature electric-field control of magnetism of heterostructures, which has inspired the development of fascinating magnetoelectric-device concepts. In this Special Topic, several articles review the past and look into the future of multiferroic physics and materials.

Single-phase multiferroics show the richness, complexity and functionality of interacting degrees of freedom (e.g. spin, charge, orbital and lattice). No unified scenario about the microscopic mechanism is yet available and the physical origins of the magnetoelectric effect could be different in single-phase multiferroics. In a review, Dong *et al.* describe the fundamental physics from a theoretical viewpoint. By taking BiFeO_3_ (best room-temperature multiferroic material so far) and *R*MnO_3_ (*R* includes rare-earth elements and yttrium) as examples, Song *et al.* review the lattice and spin dynamics and their magnetoelectric coupling from experimental and simulation perspectives. So far, several families of single-phase multiferroic materials have been found. In a review, Lu *et al.* highlight the recent progress of single-phase multiferroics and exploration of new materials such as recently emerging 2D multiferroics. Compared to single-phase multiferroics, multiferroic heterostructures typically display a larger magnetization that is more efficient to control using an electric field at room temperature. In a respective study, Hu *et al.* highlight some strategies for such electric-field control of magnetization in multiferroic heterostructures and discuss its mechanisms, current trends and future directions.

The response of multiferroic materials is also complicated by the domain structures at the nano and mesoscopic. Any improved understanding of such domain structures is necessary to enable the control of multiferroic properties. Two perspectives and two reviews in this Special Topic are devoted to this topic. In a perspective, Cheong demonstrates the intriguing topological nanoscale textures of domains/domain walls in single-phase multiferroics and how to understand the multiferroicity by the consideration of broken symmetries. Furthermore, Liu *et al.* highlight the opportunities and challenges in probing the domain structures and manipulating multiferroics on the nanoscale via a scanning probe microscopy technique. Without losing generality, Huyan *et al.* review the structures and properties of domain walls in BiFeO_3_ thin films. By using *in-situ* transmission electron microscopy, the electrical properties are correlated with the atomic-scale structures and dynamic behaviors of domain walls. Beyond this, the intriguing topological domain textures can also emerge in confined multiferroic nanostructures, as discussed in a review by Tian *et al.* highlighting recent advances in topological domain structures and the magnetoelectric effect in confined multiferroic nanostructures.

From the viewpoint of basic research, the study of multiferroics will certainly continue to grow, since the combinations and permutations of ferroic materials, polarizations and interactions are endlessly rich and productive. We hope that this Special Topic can promote further understanding of multiferroics and materials discovery. Last but not least, how to realize the practical multiferroic device applications is still very challenging, though fascinating multiferroics-based device concepts have been proposed from various viewpoints. Well beyond this progress, one can even be optimistic about an integration of multiferroicity with those emergent phenomena in condensed matters at various real and momentum space scales, such as topological quantum effects.

